# Slow‐fast atrioventricular nodal reentrant tachycardia induced by double ventricular response: A unique electrophysiological finding

**DOI:** 10.1002/joa3.12631

**Published:** 2021-09-04

**Authors:** Koji Sudo, Tetsuya Asakawa, Kazuya Nakagawa, Yuta Shimoura, Kuniyoshi Matsumura

**Affiliations:** ^1^ Department of Cardiology Yamanashi Kosei Hospital Yamanashi Japan

## Abstract

We reported a unique case of slow‐fast atrioventricular nodal reentrant tachycardia induced by double ventricular response with reproducibility.

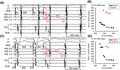

A 74‐year‐old man with sudden palpitations due to tachycardia was referred to our hospital. The baseline 12‐lead electrocardiogram (ECG) showed normal sinus rhythm without ventricular pre‐excitation and remarkable ST‐segment changes (Figure 1A). A 12‐lead ECG during narrow QRS tachycardia is shown in Figure 1B, in which the retrograde P wave was unclear. Holter monitoring showed a narrow QRS tachycardia, which occurred by atrial premature contraction and showed two diagnoses as the tachycardia mechanism: one was slow‐fast atrioventricular nodal reentrant tachycardia (AVNRT) induced by a double ventricular response (DVR; Figure 1C), and another was as junctional tachycardia (JT), as shown in Figure 1D. He had no significant comorbidities and transthoracic echocardiography showed a structurally normal heart and normal left ventricular function. We performed an electrophysiological study (EPS) with his consent. The four electrode catheters were positioned in the high right atrium (HRA), right ventricular apex (RVA), bundle of His (His), and the coronary sinus (CS). A‐H and H‐V intervals were normal at baseline. In the atrial premature stimulation (S1‐S1 600 msec), when the S1‐S2 was shortened from 360 to 340 msec, the S2‐H1 interval remained the same while the long S2‐H2 interval was revealed and supraventricular tachycardia (SVT; tachycardia cycle length = 522 msec) was induced with reproducibility (Figure 2A). Figure 2B showed AV conduction curve in atrial premature stimulation during basic atrial simultaneous pacing (ASP‐APS). The same phenomenon was shown in atrial premature stimulation during basic atrial and ventricular simultaneous pacing (AVSP‐APS; S1‐S1 600 msec, S1‐S2 360 msec) in Figure 2C. Figure 2D showed AV conduction curve in AVSP‐APS. The SVT was different from dual atrioventricular nodal non‐reentrant tachycardia, and the sequence of atrial excitement during SVT was the same as that during RVA stimulation. Upon performing entrainment pacing, burst pacing from the RVA showed entrainment of the atrium and a V‐A‐V response, which was unlikely to be diagnosed as atrial tachycardia. The orthodromic reciprocating tachycardia via a nodoventricular pathway or a nodofascicular pathway was unlikely because a single premature ventricular stimulus delivered during the His refractory period did not reset the following His activation during SVT. Moreover, JT was unlikely because a single premature atrial stimulus delivered during the His refractory period reset the following His activation during SVT.

**FIGURE 1:**
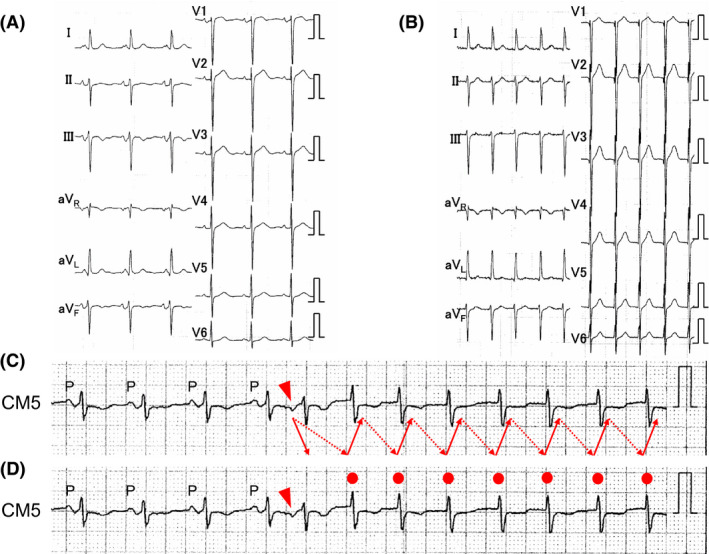
A, Twelve‐lead ECG showing sinus rhythm at 76 beats/min. B, Twelve‐lead ECG showing narrow QRS tachycardia at 124 beats/min. C, Holter monitoring extracted CM5 when SVT started. SVT that was induced by APC (red arrow head) and double ventricular response are suggestive of slow‐fast atrioventricular nodal reentrant tachycardia. Red arrows show the fast pathway and red dotted arrows show the slow pathway. D, Holter monitoring extracted CM5 when SVT started. SVT that was induced after APC (red arrow head) without jump‐up phenomenon is suggestive of junctional tachycardia. The red circles show the automaticity of the atrioventricular node or bundle of His. APC, atrial premature contraction; ECG, electrocardiogram; SVT, supraventricular tachycardia

**FIGURE 2:**
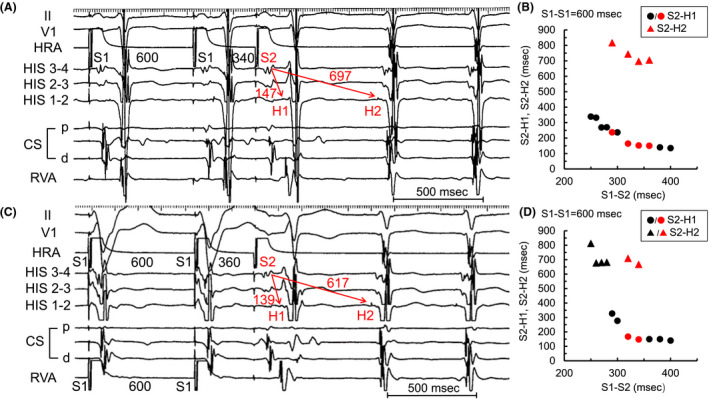
A, Intracardiac electrogram shows DVR by atrial premature stimulation during basic atrial simultaneous pacing (ASP‐APS, S1‐S1 600 msec, S1‐S2 340 msec) and SVT was induced. B, AV conduction curve in ASP‐APS. The circles denoted fast pathway conduction (S1‐H1) and the triangles indicated slow pathway conduction (S1‐H2). SVT was induced by DVR, which was shown in the red circles and red triangles. C, Intracardiac electrogram shows DVR by atrial premature stimulation during basic atrial and ventricular simultaneous pacing (AVSP‐APS, S1‐S1 600 msec, S1‐S2 360 msec) and SVT was induced. D, AV conduction curve in AVSP‐APS. SVT was induced by DVR, which was shown in the red circles and red triangles. CS, coronary sinus; DVR, double ventricular response; HRA, high right atrium; RVA, right ventricle apex; SVT, supraventricular tachycardia

We diagnosed the SVT as slow‐fast AVNRT induced by DVR. We performed radiofrequency (RF) ablation targeted to the antegrade slow pathway (SP) using anatomical guidance in Koch’s triangle (Figure 3A). A stable junctional rhythm was observed during the first RF application. After the RF ablation, AVNRT was not inducible on or off an isoproterenol infusion, and the SP disappeared (Figure 3B). The patient has remained free of any tachyarrhythmias for more than a year.

**FIGURE 3:**
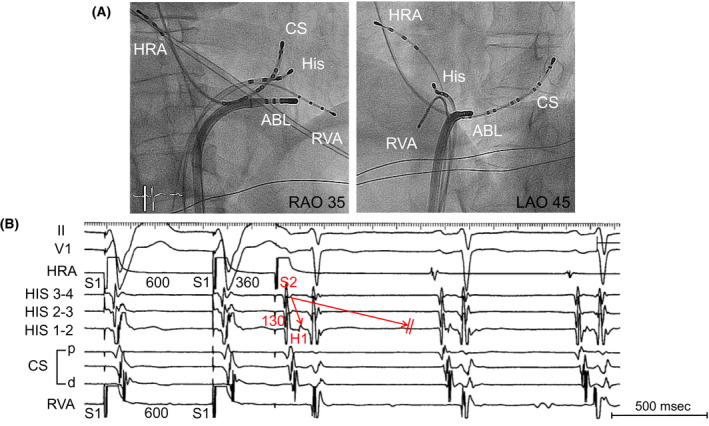
A, Fluoroscopic image shows the site of successful ablation. B, Intracardiac electrogram after radiofrequency ablation. Double ventricular response is not shown in atrial premature stimulation during basic atrial and ventricular simultaneous pacing (S1‐S1 600 msec, S1‐S2 360 msec) and supraventricular tachycardia was not inducible. ABL, ablation catheter; CS, coronary sinus; HRA, high right atrium; LAO, left anterior oblique; RAO, right anterior oblique; RVA, right ventricle apex

## 1 DISCUSSION

AVNRT is one of the most common SVT. Conversely, DVR is a relatively rare phenomenon in electrophysiological findings, which occurred with simultaneous antegrade conduction via fast and slow pathways of the atrioventricular node during one atrial excitation. DVR is first reported by Caspi G in 1979. The following conditions must be met in EPS to establish DVR:

1. There is no retrograde slow pathway, or if there is, it shows extremely weak inapparent conduction.
2. The conduction time of the antegrade SP has a length exceeding the refractory period below the lower common pathway by the preceding fast pathway.

**FIGURE 4:**
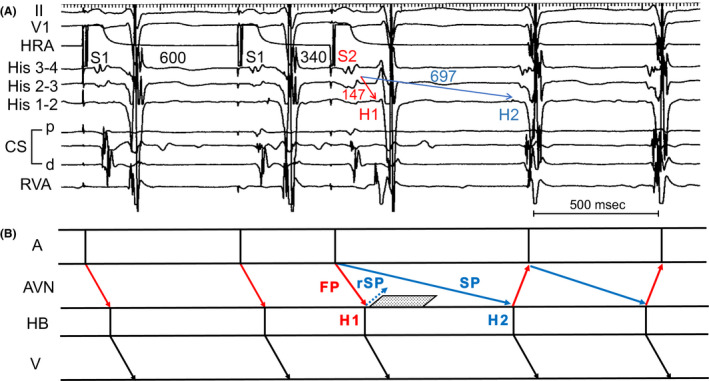
A, Intracardiac electrogram shows DVR by atrial premature stimulation during basic atrial simultaneous pacing (ASP‐APS, S1‐S1 600 msec, S1‐S2 340 msec) and supraventricular tachycardia was induced. B, Ladder diagram corresponding to ASP‐APS shown in panel A. After the last atrial pacing, DVR was observed, presumably due to the FP (red arrows) and SP (blue arrows). rSP (blue dotted arrow) shows extremely weak inapparent conduction. Black dotted area showed the refractory period below the lower common pathway by the preceding the FP. AVN, atrioventricular node; CS, coronary sinus; DVR, double ventricular response; FP, fast pathway; HRA, high right atrium; rSP, retrograde slow pathway; RVA, right ventricle apex; SP, slow pathway

We showed Intracardiac electrogram in Figure 4A when slow‐fast AVNRT was induced by ASP‐APS before RF ablation, and the corresponding ladder diagram is shown in Figure 4B to explain the mechanism of this tachycardia. Although we have observed only DVR incidentally in some cases as shown in the above conditions, this is a relatively rare case with a unique electrophysiological finding in that slow‐fast AVNRT was induced by DVR with reproducibility. In conclusion, we believe that it is important to observe DVR carefully to avoid failure in diagnosis.

## CONFLICT OF INTEREST

Authors declare no conflicts of interest for this article.

